# Transitional care decision‐making through the eyes of older people and informal caregivers: An in‐depth interview‐based study

**DOI:** 10.1111/hex.13743

**Published:** 2023-03-14

**Authors:** Lotan Kraun, Theo van Achterberg, Ellen Vlaeyen, Bram Fret, Sarah Marie Briké, Moriah Ellen, Kristel De Vliegher

**Affiliations:** ^1^ Nursing Department Wit‐Gele Kruis van Vlaanderen Brussels Belgium; ^2^ Department of Public Health and Primary Care, Academic Centre for Nursing and Midwifery KU Leuven Leuven Belgium; ^3^ Department of Health Policy and Management, Guilford Glazer Faculty of Business and Management and Faculty of Health Sciences Ben‐Gurion University of the Negev Beersheba Israel; ^4^ Department of Public Health and Primary Care, Faculty of Medicine and Life Sciences Hasselt University Hasselt Belgium; ^5^ Institute of Health Policy Management and Evaluation, Dalla Lana School of Public Health University of Toronto Toronto Canada

**Keywords:** decision‐making, empowerment, home care, nursing home, patient participation, transitional care

## Abstract

**Background:**

Older people with multifaceted care needs often require treatment and complex care across different settings. However, transitional care is often inadequately managed, and older people and their informal caregivers are not always sufficiently heard and/or supported in transitional care decision‐making.

**Objective:**

To explore older people's and informal caregivers' experiences with, views on, and needs concerning empowerment in transitional care decision‐making.

**Methods:**

A qualitative descriptive study was conducted in the TRANS‐SENIOR consortium's collaborative research using semistructured in‐depth interviews between October 2020 and June 2021 in Flanders, Belgium. A total of 29 people were interviewed, including 14 older people and 15 informal caregivers who faced a transition from home to another care setting or vice versa. Data were analysed according to the Qualitative Analysis Guide of Leuven.

**Findings:**

Five themes were identified in relation to the participant's experiences, views and needs: involvement in the decision‐making process; informal caregivers' burden of responsibility; the importance of information and support; reflections on the decision and influencing factors.

**Conclusions:**

Overall, older people and informal caregivers wished to be more seen, recognised, informed and proactively supported in transitional care decision‐making. However, their preferences for greater involvement in decision‐making vary and are affected by several factors that are both intrinsic and extrinsic. Therefore, healthcare systems might seek out age‐tuned and person‐centred empowerment approaches focusing on older people's and informal caregivers' empowerment. For future studies, we recommend developing specific strategies for such empowerment.

**Patient or Public Contribution:**

Older persons' representatives were involved in designing the TRANS‐SENIOR programme of research, including the current study. Healthcare professionals and nursing care directors were involved in the study design and the selection and recruitment of participants.

## 1 BACKGROUND

Life expectancy at birth is projected to increase significantly in Europe in the coming decades.^1^ However, people's ageing often comes with increased comorbidities, a growing demand for formal and informal care,^2^, ^3^, ^4^ high risk of hospitalisation^5^ and a transition to either short or long‐term care facilities.^6^ These transitions can have a major impact on older people's and informal caregivers’ daily lives due to feelings of confusion and powerlessness, as well as feelings of reduced autonomy when decisions are made on behalf of the older person.^7^, ^8^, ^9^, ^10^, ^11^ There is an increasing need for more integrated care to cope with such challenges and for transitional care, more specifically.^9^, ^12^, ^13^, ^14^ The American Geriatrics Society has defined transitional care as ‘a set of actions designed to ensure the coordination and continuity of healthcare as patients transfer between different locations or levels of care within the same location’.^15^

Even though the literature indicates that transitional care is crucial^15^, ^16^, ^17^ and can lead to an improvement in the quality of care,^18^, ^19^ good transitional care is not always delivered, and as a result, care transitions can also be associated with adverse consequences such as medication errors,^20^ high costs^21^ and even mortality.^22^

Research shows that factors such as information, support, and involvement in decision‐making are needed to optimise transitional care.^7^, ^8^, ^11^ Older people's and informal caregivers’ involvement in transitional care decisions is often suboptimal.^23^, ^24^, ^25^ A scoping review on older people's involvement in care transition planning and the factors guiding their decision‐making indicates that transition decisions are mainly taken by healthcare professionals, with limited or unclear involvement of older people or informal caregivers.^26^ In addition, a metasummary of literature on older people's experiences regarding participation in hospital discharge suggests that their physical and mental condition, their personal networks, national policies and norms towards shortening hospital stays, and many communication challenges all influence older people's experience with transitional care and their involvement in decision‐making.^27^ Finally, a systematic review of housing decisions amongst older people shows that factors such as feeling in control over decisions, social and support networks and personal identity can influence older people's transition decisions.^28^

According to Castro et al., empowerment is an important process through which people can obtain a greater level of involvement in the decision‐making that they desire and are enabled to achieve a greater degree of control over their health and wellbeing^29^; they also suggested antecedents for the empowerment process, such as a dialogue between healthcare providers and patients, a patient‐centred approach and the enhancement of a patients’ competences and their active participation.^29^ However, little is known about the experiences, views and needs of older people and informal caregivers regarding their involvement in transitional care decision‐making or how to best enable their empowerment in transitional care decision‐making. Hence, this study explores older people's and informal caregivers’ experiences with, views on and needs for empowerment in transitional care decision‐making in the Flemish setting (Belgium).

## 2 METHODS

### 2.1 Design

A qualitative descriptive study was conducted using semistructured in‐depth interviews.

### 2.2 Participants

This study was performed in Flanders, Belgium. A purposive sample of participants included older people and informal caregivers. Participants who faced a care transition that included a move either away from or to their home.

### 2.3 Eligibility criteria and purposive selection

Older people were eligible to participate if they: (i) were 65 years or older; (ii) experienced at least one care transition, which included a move either away from or to their home in the 6 months before the interview and (iii) spoke Dutch fluently. Informal caregivers were eligible to participate if they: (i) were an older person's primary informal caregiver; (ii) (co‐)experienced a transition pathway of their loved one in the 6 months before the interview and (ii) spoke Dutch fluently.

Older people and informal caregivers were excluded if they: (i) had a severe cognitive impairment based on a healthcare professional's clinical judgement or (ii) were in a terminal care situation.

Attention was paid to obtaining sufficient variation in gender, living arrangements (alone or with others), and dependency in activities of daily living based on a healthcare professional's clinical judgement during the recruitment phase. We also strived for variation in transitional care pathways to include pathways with (1) transitions from home to hospital to home; (2) transitions from home to hospital and then to nursing home and (3) direct transitions from home to nursing home, while allowing for the inclusion of pathways with additional or repeated transitions. The informal caregivers were not necessarily related to the older people who participated in the study. At least one informal caregiver of a person with severe dementia (who themselves were ineligible for an interview) was included for each of the three pathways described above.

### 2.4 Recruitment procedure

Concerning homecare, eligible participants were contacted by the head nurse. In the nursing homes, eligible participants were approached by either the care director or a social worker. They received an oral and written explanation of the study's rationale and aims and were asked to sign an informed consent form before the interview.

### 2.5 Data collection

Narrative data were collected from October 2020 to June 2021 using in‐depth, semistructured interviews. Two interview guides were developed (one for the interviews with older people and one for the interviews with informal caregivers) based on literature including a systematic review's findings^9^ and according to the research team's expertise. The interview guides focused on topics such as involvement, autonomy, support and control in decision‐making before, during and after the transition but also encouraged the participants to raise relevant and related topics themselves.

We planned pilot interviews with two older people. The interview guide was slightly adjusted based on the first interview. The second interview did not indicate the need for further improvement of the guide, so this person became the first inclusion for the actual study. Four researchers conducted the interviews and had previous experience in interviewing, while one researcher observed (T. v. A., S. M. B., E. V., B. F. and L. K.). The interviews were recorded and transcribed verbatim.

### 2.6 Data analysis

Data analysis was based on the Qualitative Analysis Guide of Leuven (QUAGOL).^30^ This method allowed for a continuous balance between within‐case and cross‐case analysis, as well as for the adoption of a forward‐backward approach. Six researchers conducted the analysis (L. K., T. v. A., B. F., E. V., S. M. B. and K. D. V.).

The first stage began with the preparation for the coding process and employed a case‐oriented narrative approach that enabled the researchers to identify essential and common ideas throughout the data by using the method of constant comparison. The actual coding process took place in the second stage. A thematic analysis was consistently applied to all data, based on the conceptual insights that were developed during the first stage (using qualitative research data analysis software NVivo 11).^30^

## 3 RESULTS

### 3.1 Participants

29 participants were interviewed: 14 older people and 15 informal caregivers (mean ages 83 and 62, respectively, with equal gender distribution; see Table 1 for participant characteristics). The interviews had an average duration of 50 min.

**Table 1:** Older people's and informal caregivers’ characteristics.

Characteristics	Older people (*n* = 14)	Informal caregivers (*n* = 15)
Age		
40–49		1
50–59		3
60–69	4	11
70–79	2	
80–89	4	
90–100	4	
Gender		
Female	7	8
Male	7	7
Relationship (link to an older person)		
Child	–	11
Partner	–	1
Partner's cousin	–	1
Nephew	–	1
Parent‐in‐law	–	1
Marital status		
Married	3	13
Not married (or partner deceased)	11	2
Education		
Elementary school	8	
Secondary/vocational school	5	10
Bachelor's degree		4
Master's degree (or higher)	1	1
Care pathway		
Away from home	9	10
Home to a nursing home directly	4	5
Home to the hospital and then to the nursing home	3	4
Home to the hospital to nursing home to hospital to nursing home	1	–
Home to a service flat and then to the nursing home	1	1
Away from home and returning home	5	5
Home to the hospital to home	4	3
Home to a nursing home and back home	–	1
Home to the hospital to nursing home to second nursing home to home	1	1

Participants experienced seven alternative care transition pathways, which always included a transition either away from their home or back home (see Table 1 for details).

It became clear during the interviews that three informal caregivers and two older people experienced a care transition more than 6 months previously; the interviewers asked if the experiences were still clear in their minds in all of these cases. In discussing this afterwards, the interviewers also agreed that there was no noticeable difference in the participants' recollection of events or the level of detail in how their experiences were expressed. So even though this violated the inclusion criteria, the research team decided to include these interviews in the analyses.

### 3.2 Results from the interviews

In the interviews, older people struggled to think of, clearly express and/or elaborate upon their views and needs for empowerment in transitional care decision‐making, even though the interviewers repeated and rephrased questions to explore such needs and views. Thus, an initial and significant finding is that our data contain older people and informal caregivers' experiences first and foremost.

Five main themes could be derived from the data: involvement in the decision‐making process; informal caregivers’ burden of responsibility; the importance of information and support; reflecting on the decision and influencing factors in the decision‐making process.

#### 3.2.1 Involvement in the decision‐making process

Three levels of older people's and informal caregivers’ involvement could be distinguished: (1) taking the decision autonomously and being in charge; (2) being involved and making shared decisions and (3) not being involved. At the three levels, the involvement could relate directly to the decision of whether or not to transfer from one setting to another or to how a transition was made (when, where and how). The older people's and informal caregivers’ levels of involvement in transitional care decisions were often interrelated, meaning that when an older person was highly involved in decisions, the informal caregiver was often less involved and vice versa.

##### Older people taking the decision autonomously and are in charge

Some study participants that faced a long‐term care decision reported high levels of autonomy in the decision‐making process. Some of these older people stated that the transition was their own well‐considered decision and aligned with their needs and preferences.

…I really missed her [the spouse who moved into a nursing home] and she was only gone for a week… If professional healthcare at home is no longer enough, then we had to make that decision for her… But I said no; I can't stay home alone, that is not going to happen… so I moved with her [to the nursing home]. (Older person 1, home to nursing home)

Other participants were also autonomous and were in charge of the decision, but to them, it was more of a forced decision because they felt a decline in their physical or mental condition, combined with their informal caregivers’ limited capacity, made it impossible to stay at home.

…I was absolutely unwilling to move to a nursing home. I was actually, um, not obliged but the facts, the events in the environment sent me in that direction. I wanted to resist that for as long as possible, but I had to acknowledge that it was no longer possible to stay at home. I said to myself: ‘I don't like it, but there is no other choice…’ (Older person 2, home to a nursing home)

##### Being involved and making shared decisions

Some older people and informal caregivers in this study indicated that the transition decision was a shared decision by the older person and by the informal caregiver(s) and that healthcare professionals were only a little bit involved. These participants felt involved and heard. However, some informal caregivers reported a long (and stressful) process, including several conversations and discussions, during a transition to a nursing home.

…We [the informal caregiver and the older person] have really been working for years towards the decision to move from home to a nursing home, but he always said: ‘No, when I'm 70 years old…’ and then we thought ‘Okay, we will wait’… But at a certain moment, he said… ‘it's not working anymore. I really need help’. And that was the moment that we all decided together that the move to a nursing home was the best option'. (Informal caregiver 2, home to a nursing home)

Some participants, transferring from hospital to home, indicated that they were part of the discharge decision‐making process, even though the actual decision to go home was taken by healthcare professionals. They felt that the discharge was made in dialogue with all parties involved and that they could express their preferences regarding the discharge timing.

Yes, yes. I discussed going home with the urologist… Uhm, because the day before I discussed it with him and I actually wanted to stay one more day to see how things would evolve, but then we discussed it together, that urologist is a man of dialogue. (Older person 2, home to hospital and back home)

##### Not being involved

Some participants in this study expressed not being involved in the decision‐making process; two subgroups could be distinguished.

In the first subgroup, the older people preferred relying on their informal caregivers or healthcare professionals. They did not want to be involved and believed that the (informal) caregiver would make the right decision. In the second subgroup, however, the older people and/or informal caregivers felt they had no choice and that the transitional decision was forced upon them. Older people and/or informal caregivers had to rely on the healthcare professionals’ decisions in urgent transitions, such as unplanned hospital admissions. These decisions were often made hurriedly, and some older people in this study were not even fully aware of the situation.

…My daughter was there. But even she could only follow the decision of the ambulance personnel and the doctor… She had to do what had to be done… I had no control over my own… I was really, uhm, numb. Well, I was completely gone. (Older person 4, home to hospital and then back home)

Some older people and informal caregivers in this study, involved in a transfer from the hospital to a short or long‐term care facility, felt that they were not consulted in this transitional decision. They understood and often accepted that the older person's health status made the possibility of going home impossible, but at the same time, they experienced the decision‐making process as a ‘one direction’ decision made by the hospital staff.

In the beginning you don't have a choice in that, and they send you straight from the clinic to a nursing home because they know that you can't go home from the clinic. Because I also had an operation… and now I have to accepted [living in a nursing home]. Some days it works, some days it doesn't. [silence] So I have to stay here, whether I want to or not. (Older person 10, home to hospital to a nursing home)

#### 3.2.2 Informal caregivers' burden of responsibility

Every transitional decision implied a high level of responsibility for the informal caregivers, regardless of the pathway and whether it was urgent. Responsibility was experienced in relation to finding the right information, understanding the decision's (emotional) impact, assessing all possible risks and making the best decision for everybody, especially for the older person.

Different areas of responsibility could be derived from the data, and they were all related to making the right decision at the right time and with everyone's preferred level of involvement. Most informal caregivers involved in this study were worried about their loved ones’ well‐being and whether the transitional decision was in the older person's best interest. Conversely, the feeling of responsibility was also related to practical issues, such as choosing a particular hospital or nursing home, payments for treatment or care and emptying or selling the older person's house. Some informal caregivers involved in this study indicated they also had other roles with related responsibilities, such as being someone's partner, parent and/or employer. This combination of responsibilities was experienced as a burden.

Furthermore, most informal caregivers experienced high levels of stress and burden in the transitions with low involvement from the older person because they felt they had to make a huge decision about someone else's life.

At the same time, some older people in this study also expressed concern about the well‐being of their informal caregivers and family and the fact that they do not want to be a burden on them.

I couldn't go any other way; that was the only solution. And the children… That's no longer possible; there was no other solution. No, no, it's no longer possible. You can't put the burden on the children. (Older person 12, home to hospital and back to home)

#### 3.2.3 Importance of information and support

Access to information and support from others were crucial elements in the transitional decision‐making process.

##### The importance of information

Access to information was indicated as an essential element in the transitional decision‐making process for both older people and informal caregivers in this study. This related to both the sort of information needed (medical diagnosis, treatment plan, discharge process and nursing home arrangements) and how it was delivered (face‐to‐face, by phone or digital), and in relation to the issue of timing.

Participants in this study stated they were highly dependent upon the information they received from healthcare professionals. This related to the amount and timing of the information given to them, but also to the fact that they had to ask for information and that information was not given spontaneously. This caused confusion and frustration and hindered the participants in the decision‐making process:

The biggest obstacle is that they didn't inform me. That they didn't say, ‘so or so’. Then you see that it turns out the wrong way. That she had to go back to the hospital a day and a half and two days later. (Informal caregiver 3, home to hospital and back to home)

An older person indicated that he had searched for information and treatment options. He then confronted the specialist with this information, and they discussed the best options together and in dialogue. This resulted in a fluent and pleasant transitional decision‐making process.

…I informed myself in such a way that when I talked to a doctor, I knew what was possible and what wasn't, what the alternatives were and so on. I also said to every doctor… ‘Look, things are only going to happen if you can convince me that those are the right things, and if I agree that we decide together’… (Older person 2, home to hospital and back to home)

Some study participants, when faced with a transition from home to a nursing home, felt that time was on their side. They were able to think about alternatives and visit locations. However, they indicated that information did not come to them but that they really had to make a great effort to reach out to the nursing homes for information. If they were not able to ask the right questions, then they did not receive the necessary information.

…You really have to find everything yourself. You get a whole list… of, uhm, retirement homes from, um, social services. But then you have to figure it out for yourself, so you can start calling that whole list. But you won't get that sorted out in 1‐2‐3 just like that, of course. (Informal caregiver 1, homecare, home to nursing home)

A family tie was also a crucial factor in obtaining information. One participant was the informal caregiver for both her father and his partner. She received all of the necessary information about her father's condition, treatment and health status, but she was not able to get this same information for his partner who was also hospitalised. This complicated the transitional decision‐making process.

Yes, because that's actually a totally different situation…Because at the time she [partner of the informal caregiver's father] was admitted, um…the information does not run smoothly then. Why is this the case? Because I am not family. Then you get very little information, uhm, then the moment they come home…Uhm, at that moment when you have to make a decision that's actually very difficult. (Informal caregiver 1, home to nursing home)

##### Support

Informal caregivers were the main support sources for most older people in this study. Informal caregivers help older people cope with the journey back home when they face a hospital discharge by gaining the necessary information to take part in the transitional decision‐making process for instance. In other situations, older people appreciated the efforts and support from their informal caregivers and family in arranging all of the necessary help at home because it allowed them to stay home (in nonurgent scenarios).

Informal caregivers had their own support needs that needed to be met to be able to support their loved ones. Some informal caregivers mentioned the importance of their partner, children and siblings in the decision‐making process. They carried the burden together; they also ‘ventilated’ difficulties and frustration, recharged their batteries and got advice.

The support from and the role played by healthcare professionals were not mentioned spontaneously. The older person, the informal caregivers and the family seemed to be the most important people in the transitional decision‐making process. The healthcare professionals were considered important in the process, but this was only raised when expressly asked.

Gosh yes…The social assistant actually helped us. Assistants from the residential care centre and from the hospital as well, who recommended various things to us and gave us a lot of freedom. We continued to look for things ourselves, we had a suggestion and went to the assistants… (Informal caregiver 7, home to hospital to a nursing home)

Some of the informal caregivers experienced the need for more proactive support and guidance in the transitional decision‐making process. They expressed the need for a multidisciplinary meeting and shared decision‐making after a move from hospital to home and before a move from home to a nursing home.

…Uh yes, such a person that maybe puts things in order. At the last moment or in the last few months before the decision, I really couldn't put things in order in the sense that I could look at it very rationally. I was constantly, uh, overloaded by emotions…A meeting would have led and put everything in order, and I think I would have felt supported in the sense of, Yes, I am being heard… (Informal caregiver 9, home to a nursing home)

#### 3.2.4 Reflecting on the decision

Three phases in a transitional care decision‐making process can be derived from the data: a predecision phase, the phase of the actual decision, and a postdecision phase. In the postdecision phase, participants reflected on the decision that was made. Here, participants more involved in decision‐making processes also reflected on more decision‐making aspects.

Different reflections were mentioned by participants who experienced transitions, such as from home to hospital (mostly acute) and back home, versus transitions from home to a nursing home (mostly nonacute). In the first scenario, some participants reflected on the information that they received, how they responded to treatment, and how supportive care was organised after being discharged home. These participants always had the prospect of going home. The hospital and the care facility were considered necessary (but temporary) bridges. In the second scenario, the reflection was more emotional and profound because moving to a nursing home was seen as a move towards their final destination. It was important for these participants to reflect upon the decision to relocate and whether it was the right decision in hindsight.

The older people who moved to a nursing home mostly felt relieved because everything was taken care of, and they were less alone, even though they missed their home and previous life. In these cases, the informal caregivers had more emotional reflections: doubts about whether it was the right decision, questioning if things could have been done differently, wondering if the transition could have been avoided, being concerned about the well‐being and happiness of the loved one and feelings of guilt when they did not succeed in providing their loved ones with a transfer according to their preferences. However, some informal caregivers also felt relieved when they believed that the older person was happy with the transfer/decision and was situated in a place where he/she felt safe and well‐cared for.

…It's a mixed feeling of guilt that you didn't solve it yourself, because you think: ‘I should have taken care of him myself or I should have gone to sleep there, or I should have arranged for him to stay at home in one way or another’, because that was his wish. But also some relief: he is being taken care of, it is under control, he can't go outside at night anymore, he can't do crazy things anymore. (Informal caregivers 9, home to a nursing home)

#### 3.2.5 Influencing factors

The urgency of the transition, the familiarity with the selected transition destination, a person's personality and the COVID‐19 pandemic all influenced both older people's and informal caregivers’ transitional care decision‐making experiences, views, and needs.

##### The urgency of the transition

The level of involvement in decisions was strongly influenced by the care transition's urgency. Acute situations, such as critical health problems, resulted in little time to think about alternative options, and older people were often incapable of decision‐making at such moments. Older people and their informal caregivers stated how things ‘just happened’, without a profound decision‐making process. They accepted that this transition had to happen at that time; the decisions were mostly made by the healthcare professionals in these situations.

In contrast, situations such as a nonacute move from home to a nursing home implied more time for consideration and a profound decision‐making process, exploring alternatives and preparing for the transition. Our data showed that older people and informal caregivers were more in the lead in such situations.

##### Familiarity with the destination

Being familiar with the transition destination was seen as a facilitator in the decision‐making process by both older people and informal caregivers in this study. In a move from hospital to home, older people felt safer if their home was near the hospital and if their family was close by. This was important in case something went wrong. Older people moving from home to a nursing home and who were able to stay in the same area experienced an increased feeling of confidence compared to those who had to move further away from the town or region that they had always known. ‘Well, I also think, this is in the neighbourhood… and she [the older person] also knows a lot of people here. So, she came here and immediately there were a few people she knew’ (Informal caregiver 11, home to hospital to a nursing home).

A few participants, moving from home to a nursing home with possible intermediary hospitalisation, reflected on the idea that previewing the transition experience would have helped them to better understand the concept of nursing homes. For them, being familiar with the nursing home would have facilitated the decision‐making prosses.

##### Personality

Several informal caregivers reflected on their loved one's personality as an influencing factor in the decision‐making process. Older people with a positive and optimistic personalities enabled better communication with their informal caregivers and their involvement. Other older people, perceived as stubborn or angry, were said to hinder informal caregivers' involvement and support in decision‐making.

…my father has always been like that. So, I'm used to it. That he is very dominant, uhm and that he can be aggressive. And having a conversation with my father is difficult. You can't really talk to him, also because he always knows better (laughs). (Informal caregiver 4, home to nursing home)

##### COVID‐19

The COVID‐19 pandemic influenced some participants' decision‐making processes and coloured their transition experiences. On the one hand, it hindered and disturbed some care transition decisions, but it also accelerated some of the decisions on the other. Due to the pandemic and related social or physical restrictions, some participants were confronted with unpredictable situations and experienced a shortage of information, limited contact with healthcare providers, and physical isolation both during and after the transition. Some participants reported that nursing homes had to temporarily refuse older people's admissions. This forced older people and their informal caregivers to look for other solutions, such as prolonged hospitalisation, or settling for a nursing home that was not their first choice.

Yes, the hospital's social service…and if you had any questions, she would send everything by mail then… it was almost hopeless to get an answer back… but of course that was just in the Corona time, that first wave of course. But then you're really on your own, yes… (Informal caregiver 1, home to hospital to a nursing home)

## 4 DISCUSSION

This study aimed to explore older people's and informal caregivers’ experiences with, views on and needs for empowerment in transitional care decision‐making. However, participants struggled to clearly express their views on and need for empowerment in transitional care decision‐making and elaborated on their experiences instead. Therefore, data were analysed as one data set on experiences, though views and needs may occasionally be implicitly present in the results. Based on the data, five main themes were identified: involvement in the decision‐making process; informal caregivers’ burden of responsibility; the importance of information and support; reflecting on the decision and influencing factors in the decision‐making process.

This study has identified different levels of involvement in transitional care decision‐making.

Variation in transitional care decision‐making involvement preferences was identified in this study, implying that needs will also vary in this respect. Similar variation was also reported in other literature^9^, ^11^, ^31^ highlighting the older people's and informal caregivers’ involvement needs and experiences. Further, a systematic review by Murray et al.^32^ identified levels of transitional care involvement ranging from ‘non‐involvement’ to ‘autonomous acting’.

The importance of information was indicated as an essential aspect in the transitional decision‐making process for both older people and informal caregivers in this study. In line with this, a systematic review reporting on the transitional care experiences of older people emphasised the need for adequate information, discussion of uncertainties and knowledge exchange as essential parts of older people's participation in the transitional care process.^27^ Also, most of this study's participants expressed the importance of *timely* information. With a view to empowerment in decision‐making, information was also reported as a pivotal element by Castro et al.^29^ Furthermore, the TRANSCIT model for transitional care as developed by Groenvynck et al.^10^ also highlights the need for person‐centred information, meaning that the older person and the informal caregiver both have to receive information that is adjusted to both their personals needs and transition phase. These findings and ours show the need for more personalised solutions and support that suits the transition ‘story’ of the older person and his/her informal caregivers.

Apart from information, respondents in our study stressed the need for practical and/or emotional support in the decision‐making process. This finding is mirrored in two recent reviews of the literature^9^, ^26^ that underline the importance of family support for informal caregivers and the informal caregivers' support for their loved ones during care transitions. Our study adds the importance of informal caregivers' wishes for timelier multidisciplinary follow‐up and guidance in transitional care decision‐making.

Our study's informal caregivers experienced a high level of responsibility, often resulting in feelings of burden. The fact that 11 of the 15 informal caregivers in this study were the children of the older person may imply a high level of direct responsibility and commitment and thus burden. Yet this finding was also reflected in two studies and in one systematic review, in which results indicated informal caregivers’ worries about whether or not they were making the right decisions for their loved ones, as well as feelings of ambivalence, such as guilt versus relief after a nursing home admission.^33^, ^34^, ^35^

According to our findings, experiences in transitional care decision‐making and involvement preferences and engagement, in particular, might be explained by the transition's urgency. Older people (and their informal caregivers) who faced an acute transition were often less active or were even totally uninvolved in the decision‐making prosses. Other influencing factors were personalities, familiarity with the transition destination and the COVID‐19 pandemic, which sometimes hindered and sometimes accelerated some of the decisions.

### 4.1 Strengths and limitations

The study's main strength lies in the inclusion of both older people's and informal caregivers' perspectives, as well as the provision of several care transition pathways. This allowed us to capture a great diversity of perspectives. Another strength lies in the rigourous analysis of the data. The QUAGOL^30^ approach allowed the research team to go in‐depth with the whole data set in a forward‐backward analysis.

A limitation of this study is that in 5 cases (out of 29), the inclusion criterion for ‘time since transition’ was not met (transition was longer than 6 months ago). The data were still included, however, and these did not stand out as really different from the other interviews because of the rich information in those interviews in relation to the research question. A second major limitation is that depth was not reached equally well in all interviews. While all participants could provide a clear account of events, the interviewers were not always able to get the respondents to fully reflect on events or to translate experiences into views and, especially, into needs. Consequently, our results do not contain a clear overview of needs, although implicit needs may stand out from what was important to our participants in some cases (e.g., the importance of information). Finally, not all potential transitional pathways were included (e.g., no transitions to/from psychiatric hospitals, rehabilitation centres or hospice care). This limits our findings’ generalisability.

## 5 CONCLUSIONS

Overall, older people's and informal caregivers’ experiences indicate their need to be proactively recognised, informed, and supported in transitional care decision‐making. Involvement in decision‐making is crucial to ensuring high quality in transitional care. However, preferences for involvement vary and are affected by several intrinsic and extrinsic factors. Therefore, healthcare systems might seek out age‐tuned and person‐centred approaches focusing on older people's and informal caregivers’ empowerment. This can enable older people and informal caregivers to achieve a greater influence over their transition decisions by encouraging them to gain more control over issues they define as important.

## 6 IMPLICATIONS FOR FUTURE CARE AND RESEARCH

The results of this study indicate a need for the consideration of a more person‐ (or family‐) centred approach to better understand persons’ decision‐making and their transition circumstances. Such person‐centredness is often a prerequisite for empowerment.^29^ In addition, our results point to the importance of emotional and practical support and guidance for informal caregivers, even after a care transition is made. Though our study reports on data for alternative care transitions, findings relate to older people and informal caregivers in Flanders (Belgium) only. However, because our findings are largely reflected by other studies from different countries, we can recommend healthcare professionals internationally consider a setting‐tuned and tailored approach for empowering older people and informal caregivers in transitional care decision‐making.

For future research, we recommend focusing on the development and evaluation of contextualised methods by which to empower both older people and informal caregivers in transitional care decision‐making.

## AUTHOR CONTRIBUTIONS

*Conception and design*: Lotan Kraun, Theo van Achterberg, Moriah Ellen and Kristel De Vliegher. *Data collection*: Lotan Kraun, Theo van Achterberg, Ellen Vlaeyen, Bram Fret and Sarah Marie Briké. *Data analysis*: Lotan Kraun, Theo van Achterberg, Ellen Vlaeyen, Bram Fret, Sarah Marie Briké and Kristel De Vliegher. *Manuscript writing*: Lotan Kraun. All authors provided comments on the manuscript drafts. All authors read and approved the final manuscript.

## CONFLICT OF INTEREST STATEMENT

The authors declare no conflict of interest.

## ETHICS STATEMENT

The study protocol was approved by the Ethics Committee for Research UZ/KU Leuven Medical Ethics Committee (protocol number S64423). We obtained verbal and written consent before each interview. Data were pseudoanonymised for privacy and confidentiality reasons. Since the target group consists of vulnerable persons, sufficient attention was paid to supporting the older people and informal caregivers during the interviews by clarifying expectations before the interview. This allowed participants sufficient time to formulate their answers, speaking loud enough, reformulating the question when necessary and providing postinterview debriefing time ensured that the person was satisfied with the interview and that their concerns and well‐being were sufficiently addressed. The fact that participants could end the interview at any time was also stressed. Verbal and written consent was obtained before each interview.

## Data Availability

The data that support this study's findings are available from the research team upon reasonable request.
